# IL-31 is crucial for induction of pruritus, but not inflammation, in contact hypersensitivity

**DOI:** 10.1038/s41598-018-25094-4

**Published:** 2018-04-27

**Authors:** Ayako Takamori, Aya Nambu, Keiko Sato, Sachiko Yamaguchi, Kenshiro Matsuda, Takafumi Numata, Takeru Sugawara, Takamichi Yoshizaki, Ken Arae, Hideaki Morita, Kenji Matsumoto, Katsuko Sudo, Ko Okumura, Jiro Kitaura, Hiroshi Matsuda, Susumu Nakae

**Affiliations:** 1 Laboratory of Systems Biology, Center for Experimental Medicine and Systems Biology, The Institute of Medical Science, The University of Tokyo, Tokyo, 108-8639 Japan; 2 Atopy Research Center, Juntendo University, Tokyo, 113-8412 Japan; 3 Laboratory of Veterinary Molecular Pathology and Therapeutics, Division of Animal Life Science, Institute of Agriculture, Tokyo University of Agriculture and Technology, Tokyo, 183-8509 Japan; 4 Department of Dermatology, Tokyo Medical University, Tokyo, 160-0023 Japan; 5 Department of Cardiovascular Surgery, Saitama Medical Center, Jichi Medical University, Saitama, 330-8503 Japan; 6 Department of Allergy and Clinical Immunology, National Research Institute for Child Health and Development, Tokyo, 157-8535 Japan; 7 Animal Research Center, Tokyo Medical University, Tokyo, 160-8402 Japan; 8 Precursory Research for Embryonic Science and Technology, Japan Science and Technology Agency, Saitama, 332-0012 Japan

## Abstract

IL-31, which is a member of the IL-6 family of cytokines, is produced mainly by activated CD4^+^ T cells, in particular activated Th2 cells, suggesting a contribution to development of type-2 immune responses. IL-31 was reported to be increased in specimens from patients with atopic dermatitis, and IL-31-transgenic mice develop atopic dermatitis-like skin inflammation, which is involved in the pathogenesis of atopic dermatitis. However, the role of IL-31 in development of contact dermatitis/contact hypersensitivity (CHS), which is mediated by hapten-specific T cells, including Th2 cells, is not fully understood. Therefore, we investigated this using IL-31-deficient (*Il31*^−/−^) mice, which we newly generated. We demonstrated that the mice showed normal migration and maturation of skin dendritic cells and induction of hapten-specific T cells in the sensitization phase of FITC-induced CHS, and normal induction of local inflammation in the elicitation phase of FITC- and DNFB-induced CHS. On the other hand, those mice showed reduced scratching frequency and duration during FITC- and/or DNFB-induced CHS. Our findings suggest that IL-31 is responsible for pruritus, but not induction of local skin inflammation, during CHS induced by FITC and DNFB.

## Introduction

IL-31, which is a member of the IL-6 family of cytokines, is produced mainly by activated CD4^+^ T cells, in particular activated Th2 cells, mast cells, macrophages and dendritic cells^1–4^.Human and mouse IL-31 genes are located on chromosome 12q24.31 and chromosome 5, respectively, and their amino acid sequences show 31% homology^1^. Expression of IL-31 mRNA was reported in various human tissues, such as the testis, bone-marrow, skeletal muscle and kidney^1^. The receptor for IL-31 is a heterodimer, consisting of IL-31 receptor A (IL-31 RA) and oncostatin M receptor (OSMR)^1^. In humans and/or mice, IL-31 RA mRNA expression is found in various tissues, such as the testis, bone marrow, skin and dorsal root ganglia, and in various cells, such as activated monocytes, macrophages, dendritic cells (DCs), eosinophils, basophils and keratinocytes, while OSMR mRNA is broadly expressed in many tissues^5–7^. In cultures of various human cell lines such as keratinocytes^1^, intestinal and bronchial epithelial cell lines^8,9^, DCs^7^, monocytes and macrophages^10^, IL-31 has been shown to induce expression of such cytokines as IL-6, IL-8 and/or TNF and such chemokines as CCL2, CCL4, CCL5, CCL17 and/or CCL22.

IL-31 has been implicated to be involved in the pathogenesis of such allergic disorders as rhinitis, asthma and dermatitis^5^. Indeed, increased levels of IL-31 were observed in nasal secretions of patients with allergic rhinitis^11^. IL-31 levels in sera or PBMCs were significantly increased in patients with asthma^12^, and IL-31 can activate human bronchial epithelial cell lines, as described above. However, ovalbumin-induced airway inflammation developed normally in mice treated with anti-IL-31RA neutralizing Ab^13^, suggesting that IL-31 is not crucial for induction of allergic airway inflammation in that model.

In regard to skin diseases, elevated levels of IL-31 were observed in such specimens as plasma, sera and/or cutaneous biopsies from patients with atopic dermatitis^14,15^ or contact dermatitis^16^. In particular, IL-31 was implicated in itching by inducing activation of sensory nerve cells^5^. In support of that notion, transgenic mice (Tg mice), which express IL-31 systemically or specific for lymphocytes, spontaneously develop skin inflammation resembling human atopic dermatitis and show severe scratching behavior accompanied by exfoliation of epidermis^1^. Expression of IL-31 mRNA was increased in inflamed lesions of skin from mice that developed allergic dermatitis resembling human atopic dermatitis^17^. Administration of anti-IL-31 or anti-IL-31RA neutralizing Ab resulted in attenuation of scratching behavior, but not the severity of skin inflammation, in that mouse model^18,19^ or in patients with atopic dermatitis^20^. On the other hand, the role of IL-31 in the development of contact dermatitis/contact hypersensitivity (CHS) is not well understood. CHS is a T-cell-mediated cutaneous allergic response^21^. Several studies using type-2-cytokine-deficient mice showed that type-2 cytokines are important for the development of CHS (reviewed in^22^), suggesting that IL-31 may be involved in induction of CHS. *Il31ra*^−/−^ mice showed enhanced type-2 immune responses due to hyperresponsiveness to OSM by increased formation of OSM receptors (the complex of gp130 and OSMR) rather than due to failure to form IL-31 receptors (the complex of IL-31RA and OSMR)^13^. Accordingly, we deemed that murine strain to be unsuitable for examining the role of IL-31 in CHS, and we instead used IL-31-deficient (*Il31*^−/−^) mice, which we newly generated.

## Results

### Generation of *Il31*^−/−^ mice

**Figure 1:**
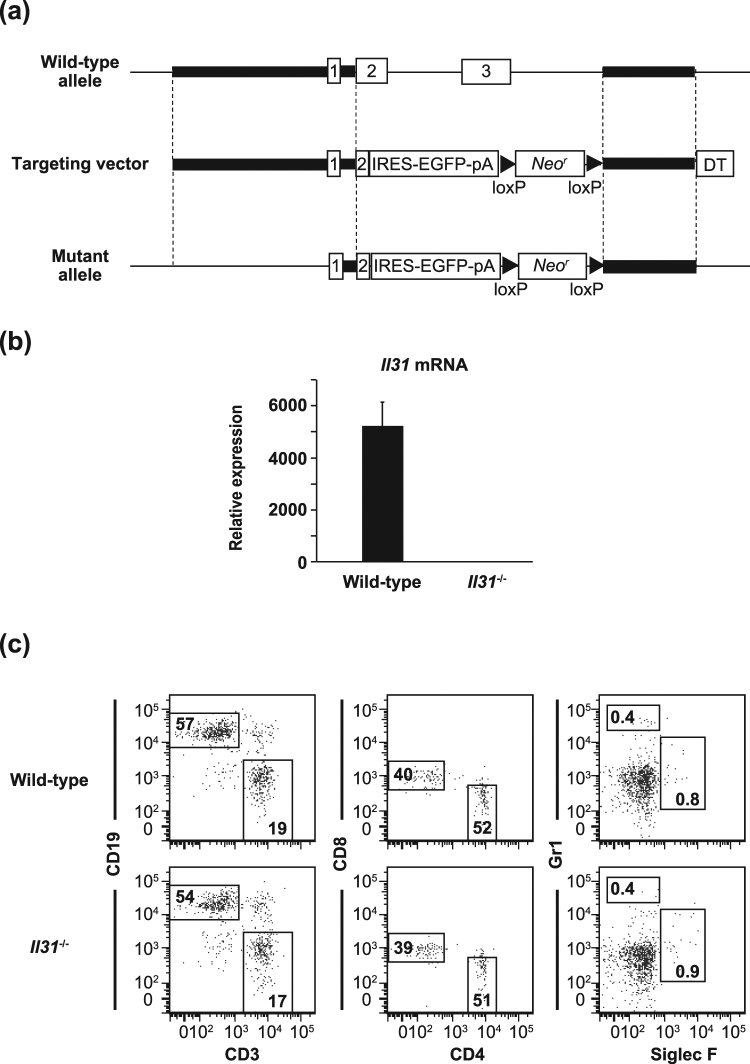
Generation of *Il31*^−/−^ mice. (**a**) IL-31 gene targeting strategy. The region containing a part of exon 2 and exon 3 of *Il31* was replaced with a cassette consisting of IRES-EGFP and a neomycin resistance gene (Neo^r^), flanked by *loxP* sequences. (**b**) Expression of *Il31* mRNA in lung tissue from wild-type (n = 8) and *Il31*^−/−^ (n = 8) mice by qPCR. The data show the mean + SEM. (**c**) Leukocyte profiles in the spleen from wild-type and *Il31*^−/−^ mice (n = 3). The percentages of CD19^+^ CD3^−^ and CD19^−^ CD3^+^ cells and of Gr1^+^ Siglec F^−^ and Gr1^-^ Siglec F^+^ cells in 7-AAD-negative spleen cells, and the percentages of CD8^+^ CD4^−^ and CD8^−^ CD4^+^ cells in 7-AAD-negative CD3^+^ spleen cells are shown. Representative flow cytometry data are shown.

*Il31ra*^−/−^ mice were reported to have not only loss of IL-31 function but also elevated responsiveness to OSM^13^, suggesting that it is necessary to elucidate the exact roles of IL-31 *in vivo* by using *Il31*^−/−^ mice rather than *Il31ra*^−/−^ mice. Therefore, we newly generated *Il31*^−/−^ mice by replacing *Il31* genes with a cassette consisting of IRES-EGFP and a neomycin resistance gene, flanked by *loxP* sequences (Fig. 1a). *Il31*^−/−^ mice were born at the expected Mendelian ratio, fertile, and did not show any gross phenotypic abnormalities under specific-pathogen-free housing conditions (data not shown). Expression of *Il31* mRNA was below the limit of detection by qPCR in the lung and in PMA-ionomycin-stimulated spleen cells of *Il31*^−/−^ mice (Fig. 1b and data not shown). No apparent abnormalities were found in the proportions of immune cells in the thymus, LNs, spleen and/or bone marrow between wild-type and *Il31*^−/−^ mice (Fig. 1c, and data not shown).

### IL-31 is not essential for skin DC function or hapten-specific LN cell response in the sensitization phase of CHS

**Figure 2:**
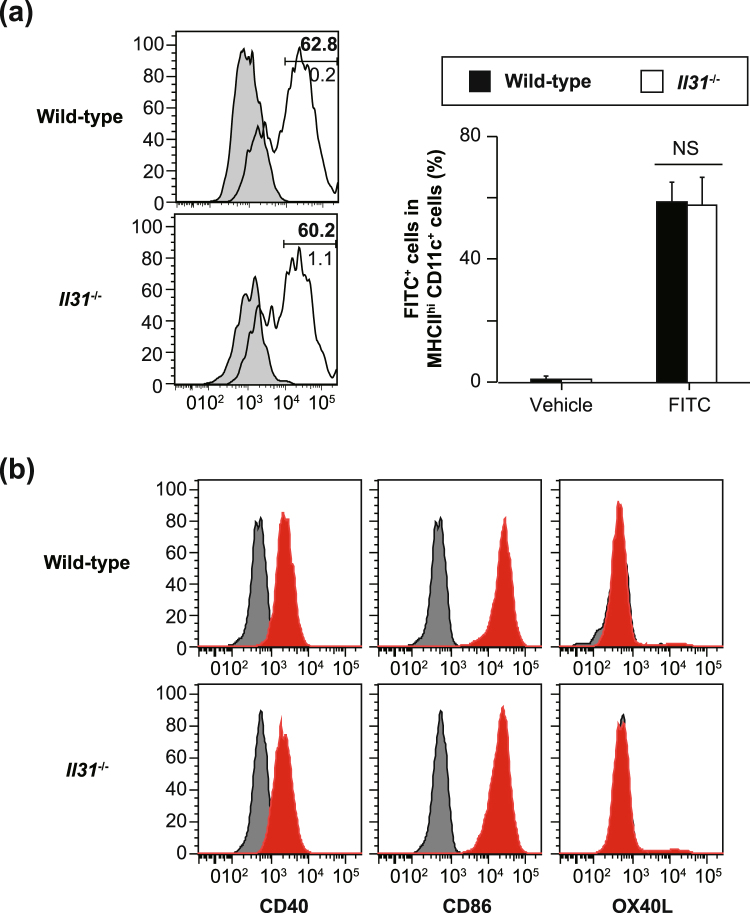
Normal skin DC function in *Il31*^−/−^ mice. (**a**) The proportion of FITC-positive cells among 7-AAD-negative MHC class II^hi^ CD11c^+^ cells in draining LNs from wild-type (n = 4) and *Il31*^−/−^ (n = 4) mice 24 hours after sensitization with FITC or vehicle alone was determined by flow cytometry. Representative flow cytometric data are shown. Shaded areas = LNs obtained from the vehicle-treated left ear (lower number [%]), and solid lines = LNs obtained from the FITC-treated right ear (upper number [%]). The data show the mean + SEM. (**b**) The expression levels of CD86, CD40 and OX40L on 7-AAD-negative MHC class II^hi^ CD11c^+^ FITC-positive cells in draining LNs from wild-type and *Il31*^−/−^ mice 24 hours after sensitization with FITC or vehicle alone were determined by flow cytometry. Representative flow cytometric data are shown for wild-type (n = 6) and *Il31*^−/−^ (n = 4) mice. Shaded areas = isotype-matched control Ig staining, and solid lines = specific mAb staining.

Treatment of mice that developed atopic dermatitis-like skin inflammation with anti-IL-31 or anti-IL-31RA neutralizing Ab resulted in attenuation of scratching behavior, but not the severity of skin inflammation^18,19^. On the other hand, the role of IL-31 in the development of CHS is not well understood. After epicutaneous exposure to haptens, skin DCs capture the haptens and migrate from the skin into draining LNs, followed by antigen presentation to naïve T cells to induce hapten-specific T cells^21^. To elucidate the role of IL-31 in skin DC migration and maturation, wild-type and *Il31*^−/−^ mice were treated epicutaneously with FITC. Twenty-four hours later, the proportion of FITC-positive cells among MHC class II^hi^ CD11c^+^ cells in draining LNs and their expression of co-stimulatory molecules were determined by flow cytometry. The proportions of FITC-positive cells were comparable between the wild-type and *Il31*^−/−^ mice (Fig. 2a). Such co-stimulatory molecules as CD86, CD40 and OX40L on the FITC-positive cells were also similarly expressed in the *Il31*^−/−^ and wild-type mice (Fig. 2b).

**Figure 3:**
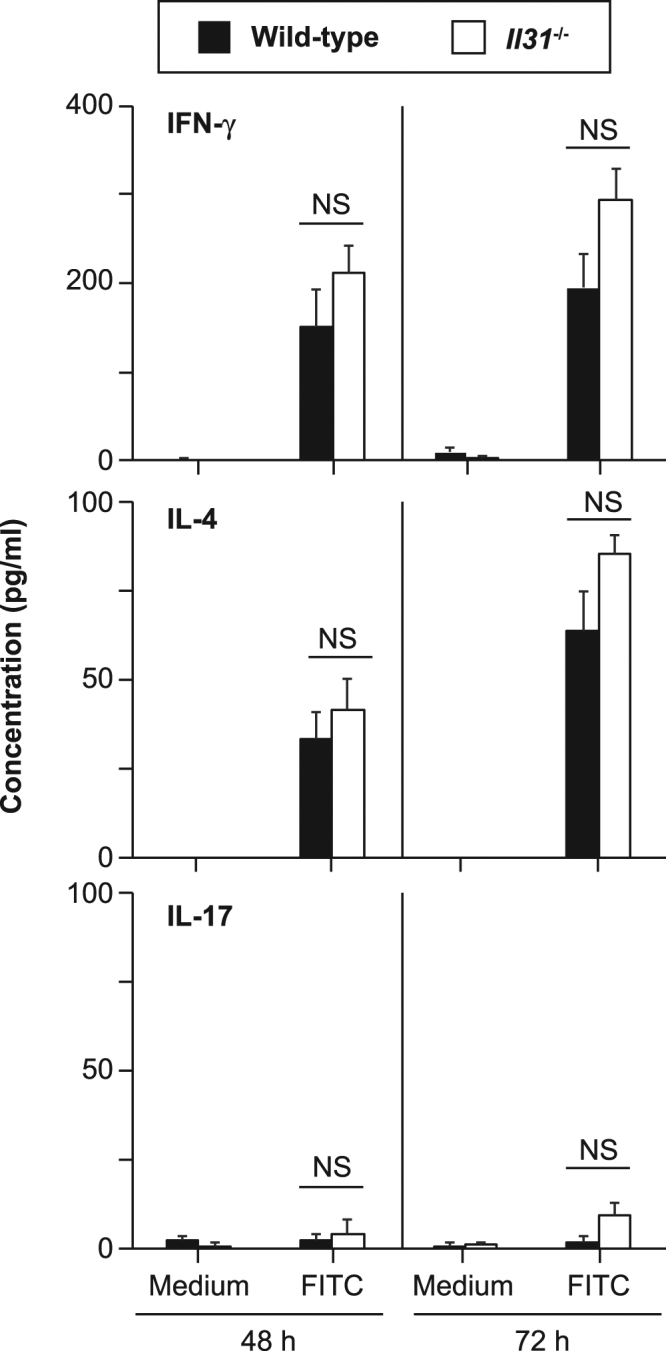
Normal hapten-specific LN cell responses in *Il31*^−/−^ mice. (**a**) LN cells from FITC-sensitized wild-type (n = 5) and *Il31*^−/−^ (n = 5) mice were cultured in the presence and absence of 40 μg/ml FITC for 48 and 72 hours. The levels of IFN-γ, IL-4 and IL-17 in the culture supernatants were determined by ELISA. The data show the mean + SEM.

We next elucidated whether the lack of IL-31 influenced hapten-specific T-cell induction in the sensitization phase of CHS. After epicutaneous sensitization of *Il31*^−/−^ and wild-type mice with FITC, draining LN cells were harvested and cultured in the presence and absence of FITC. After 48 and 72 hours of culture, the levels of IFN-γ, IL-4 and IL-17 in the culture supernatants were comparable in the two mouse strains (Fig. 3). Therefore, these observations suggest that, for the sensitization phase of CHS, IL-31 is not essential for such skin DC functions as migration, maturation and hapten-specific T-cell induction.

### IL-31 is involved in pruritus, but not inflammation, during CHS

**Figure 4:**
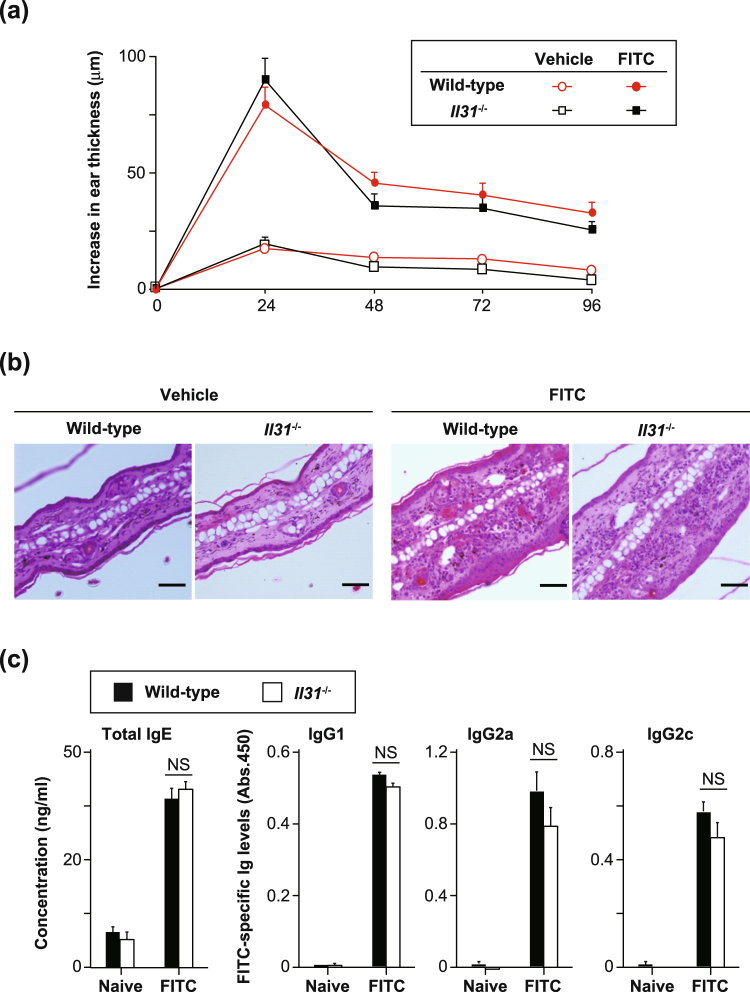
IL-31 is not essential for skin inflammation during FITC-induced CHS. (**a**) Wild-type (n = 47) and *Il31*^−/−^ (n = 41) mice were epicutaneously sensitized and challenged with FITC. The ear skin thickness of the mice was measured at the indicated time points after challenge with FITC or vehicle alone. The data show the mean + SEM. (**b**) Sections of ear skin from wild-type and *Il31*^−/−^ mice at 24 hours after challenge with FITC or vehicle alone were stained with hematoxylin and eosin. Representative data are shown. Scale bar = 50 μm. (**c**) Ninety-six hours after challenge with FITC, sera were collected from wild-type (n = 5 [naïve], and n = 21 [FITC]) and *Il31*^−/−^ (n = 5 [naïve], and n = 15 [FITC]) mice. The levels of FITC-specific IgG1, IgG2a and IgG2c and total IgE in the sera were determined by ELISA. The data show the mean + SEM.

**Figure 5:**
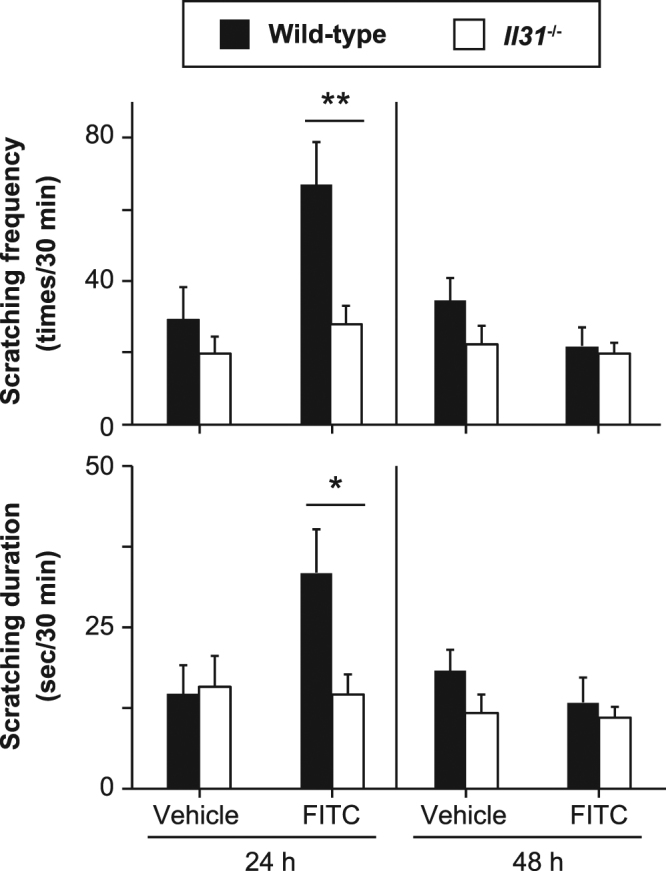
IL-31 is important for pruritus during FITC-induced CHS. (**a**) Scratching behavior (number of times and duration) in wild-type (n = 12–14) and *Il31*^−/−^ (n = 12–15) mice was assessed at 24 and 48 hours after challenge with FITC or vehicle alone. The data show the mean + SEM. *p < 0.05 and **p < 0.01.

**Figure 6:**
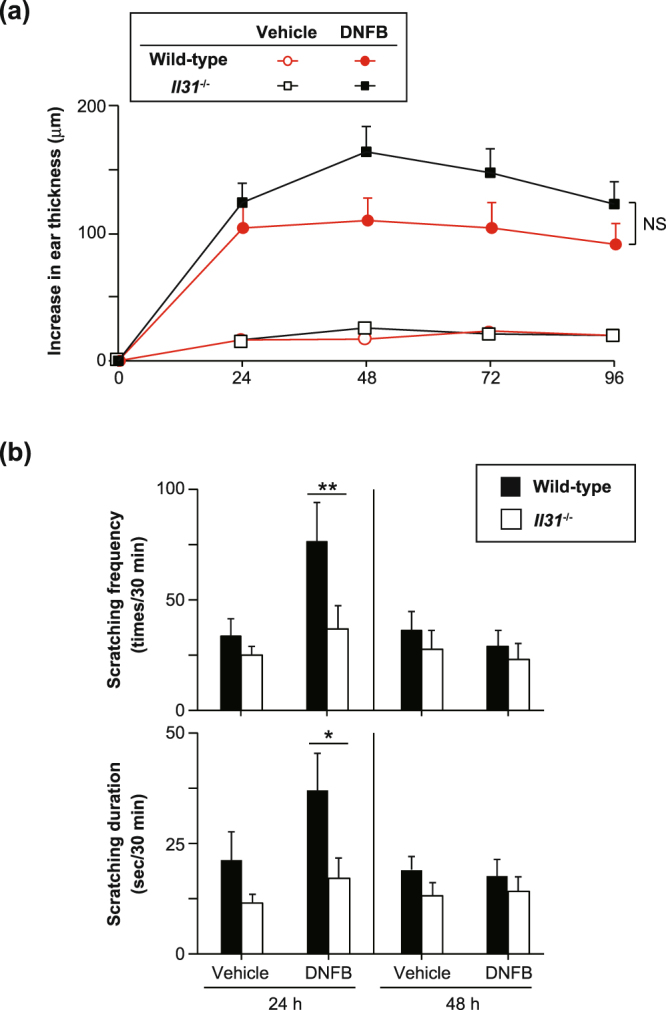
IL-31 is important for pruritus, but not skin inflammation, during DNFB-induced CHS. (**a**) Wild-type (n = 12) and *Il31*^−/−^ (n = 12) mice were epicutaneously sensitized and challenged with DNFB. The ear skin thickness of the mice was measured at the indicated time points after challenge with DNFB or vehicle alone. The data show the mean + SEM. (**b**) Scratching behavior (number of times and duration) in wild-type (WT; n = 8–13) and *Il31*^−/−^ (n = 8–12) mice was assessed at 24 and 48 hours after challenge with DNFB or vehicle alone. The data show the mean + SEM. *p < 0.05 and **p < 0.01.

**Figure 7:**
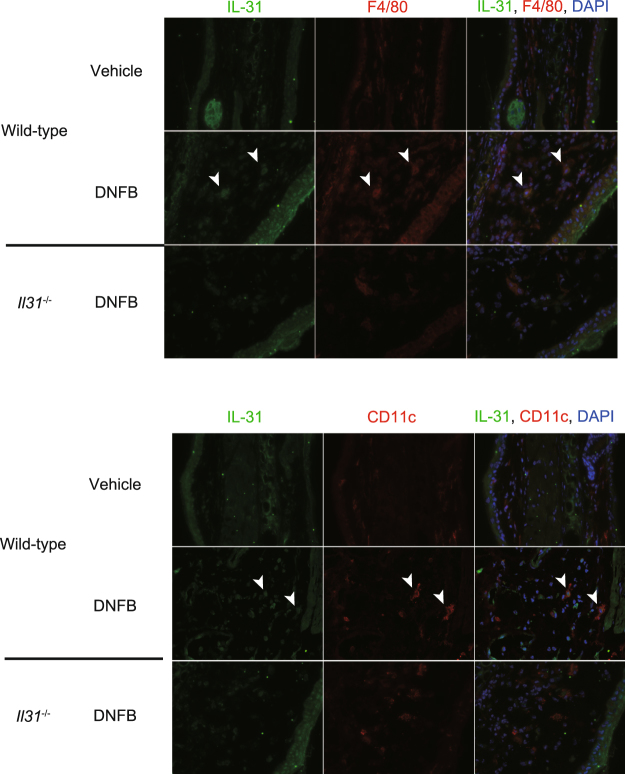
IL-31 is produced by macrophages and dermal DCs in the skin during DNFB-induced CHS. Wild-type and *Il31*^−/−^ mice were epicutaneously sensitized and challenged with DNFB. 24 h after the epicutaneous challenge with DNFB or vehicle alone, the ear skin was collected. IL-31 expression in sections of the ear skin was detected by immunohistochemistry. Arrowhead(s) = representative IL-31^+^ F4/80^+^ cells or IL-31^+^ CD11c^+^ cells.

We next investigated whether IL-31 is involved in the pathogenesis of FITC-induced CHS. The degree of skin inflammation was assessed by measuring the thickness of the ear skin after challenge with FITC. The ear thickness was comparable between wild-type and *Il31*^−/−^ mice at the indicated time points after the initial epicutaneous application of FITC (Fig. 4a). The degree of skin inflammation assessed by histological analysis was also similar between the wild-type and *Il31*^−/−^ mice at 24 hours after challenge with FITC or vehicle (Fig. 4b). We also used ELISA to examine the levels of FITC-specific IgG subsets and total IgE in sera from naïve and FITC-treated mice. After FITC challenge, the levels of FITC-specific IgG1, IgG2b and IgG2c and total IgE in sera from *Il31*^−/−^ mice increased similarly to those from wild-type mice (Fig. 4c). On the other hand, the scratching frequency and duration were significantly decreased in *Il31*^−/−^ mice compared with wild-type mice at 24 hours after FITC challenge (Fig. 5). Similar phenotypes were also observed for *Il31*^−/−^ mice during DNFB-induced CHS. Although the ear thickness was slightly, but not significantly, increased in *Il31*^−/−^ mice compared with wild-type mice at the indicated time points after the initial epicutaneous application of DNFB (Fig. 6a), the scratching frequency and duration were significantly decreased in *Il31*^−/−^ mice compared with wild-type mice at 24 hours after DNFB challenge (Fig. 6b). GFP^+^ cells were hardly detected in LNs from *Il31*^gfp/+^ mice during DNFB-induced CHS (data not shown). We found that a part of F4/80^+^ and CD11c^+^ cells, but not CD3^+^ cells, Gr1^+^ cells and tryptase^+^ cells (data not shown), were producers of IL-31 in skin from wild-type mice, but not *Il31*^−/−^ mice, at 24 h after DNFB challenge (Fig. 7). The signals in keratinocytes from wild-type mice were due to non-specific binding of the anti-IL-31 Ab, since they were detected even in *Il31*^−/−^ mice (Fig. 7). These observations suggest that IL-31 is important for induction of pruritus, but not inflammation, in CHS induced by FITC and DNFB.

## Discussion

IL-31 is preferentially produced by Th2 cells in Th subsets, and excessive IL-31 production in IL-31 Tg mice resulted in development of dermatitis due to enhancement of type–2 immune responses^1^. By contrast, *Il31ra*^−/−^ mice showed enhanced type–2 immune responses during nematode infection^23^, suggesting that IL-31R signaling has a suppressive role in type-2 immune responses. Regarding the apparent discrepancies in phenotype between IL-31 Tg mice^1^ and *Il31ra*^−/−^ mice^23^, the latter were reported to be hyperresponsive to OSM due to increased formation of OSM receptors (gp130 and OSMR) caused by the lack of IL-31R (IL-31RA and OSMR), resulting in OSM-mediated suppression of type–2 immune responses^13^. Therefore, to investigate the precise role of IL-31 *in vivo*, we newly generated *Il31*^−/−^ mice for the present study. Using those mice, we demonstrated for the first time that IL-31 is important for induction of pruritus, but not inflammation, in CHS induced by FITC and DNFB.

IL-31 is considered to be involved in the pathogenesis of type–2 cytokine-associated allergic disorders such as rhinitis, asthma and dermatitis^5^. In support of that notion, the levels of IL-31 were increased in patients with allergic rhinitis^11^, asthma^12^ and atopic dermatitis^14,15^. DNFB- and/or FITC-induced CHS was attenuated in *Il4*^−/−^ mice^24,25^ and/or *stat6*^−/−^ mice^26^, suggesting that type-2 cytokines are important for induction of DNFB- and/or FITC-induced CHS. In addition, the levels of IL-31 were also elevated in patients with contact dermatitis^16^. These observations suggest that IL-31 may be involved in induction of type-2-cytokine-associated CHS. However, we demonstrated that *Il31*^−/−^ mice showed normal migration and maturation of skin DCs and induction of hapten-specific T cells in the sensitization phase of FITC-induced CHS, and normal induction of local inflammation in the elicitation phase of FITC- and DNFB-induced CHS, indicating that IL-31 is not essential for induction of skin inflammation during FITC- or DNFB-induced CHS.

As a unique function of IL-31, it is known to be involved in itching via signal activation of IL-31 receptors on sensory nerve cells^5,27^. Indeed, IL-31 Tg mice show severe scratching behavior accompanied by exfoliation of epidermis^1^. Administration of anti-IL-31 or anti-IL-31RA neutralizing Ab resulted in attenuation of scratching behavior, but not the severity of skin inflammation, in mice that developed atopic dermatitis-like skin inflammation^18,19^. Consistent with this, we showed that *Il31*^−/−^ mice had reduced scratching frequency and duration in FITC- and/or DNFB-induced CHS, indicating that IL-31 is responsible for pruritus in the setting. However, the underlying mechanism of this remains unclear and should be further investigated in future studies.

In summary, we demonstrated here that IL-31 is involved in pruritus reactions without affecting induction of local skin inflammation in CHS.

## Methods

### Mice

C57BL/6 N wild-type mice were obtained from Japan SLC, Inc. (Hamamatsu, Japan). *Il31*^−/−^ mice on the C57BL/6 N background were generated as follows. The *Il31* gene was disrupted by replacement of the region from a part of exon 2 to a part of the intron behind exon 3 with a cassette consisting of IRES-EGFP and a neomycin resistance gene, flanked by *loxP* sequences (Fig. 1a). Homologous regions were amplified by PCR using the following primers: 5′-CAACCTATTCCTAGTTCCCTCACC-3′ and 5′-AAAGCAGCACCAGGGTAGGCTTCG-3′ to generate a 6-kb fragment, and 5′- TGAATGAGGGGAACAGAAAATTACC-3′ and 5′-TGATGAATAATGATATTCCTTACC-3′ to generate a 2-kb fragment. The targeting vector was electroporated into C57BL/6 N ES cells (EGR-101, kindly provided by Dr. Masaru Okabe, Osaka University). Male chimeric mice were obtained from three distinct targeted clones and mated with C57BL/6 N female mice. Genotyping of *Il31*^−/−^ mice was performed by PCR using the following primers: common 1 (5′-GACAACCCTTTATATATTGCCTGG-3′), WT 1 (5′-GCTTCCATAGCTGGCTTTATATCG-3′) and IL-31 KO (5′-CGACACCGGCCTTATTCCAAGCGG-3′). The common 1 and WT 1 primers were used for detection of wild-type alleles (~550 bp), and the common and IL-31 KO primers were used for detection of mutant alleles (~390 bp). For generation of *Il31*^*egfp/egfp*^ mice, a plasmid carrying Cre cDNA (pCAG-Cre, kindly provided by Dr. Jun-ichi Miyazaki, Osaka University) was injected into fertilized eggs from *Il31*^−/−^ mice to deplete the neomycin resistance gene, and it was flanked by *loxP* sequences. The eggs were then transferred into pseudopregnant female mice. Genotyping of *Il31*^*egfp/egfp*^ mice was performed by PCR using the following primers: common 2 (5′-TGGGAAGATACATGAAACCAATCC-3′), WT 2 (5′-CACTGGCTGCTCTTCCAGAGGACC-3′) and Neo^r^ (5′-GACGTGCTACTTCCATTTGTCACG-3′). The common and WT primers were used for detection of wild-type alleles (~493 bp), and the common and Neo^r^ primers were used for detection of mutant alleles (no band). All mice were housed in a specific-pathogen-free environment at The Institute of Medical Science, The University of Tokyo. The animal protocol for experiments was approved by the Institutional Review Board of the Institute (A11-28 and A14-10), and all experiments were conducted according to the ethical and safety guidelines of the Institute.

### Quantitative PCR

Total RNA was prepared from lungs using Sepasol (Nacalai Tesque, Inc.) and treated with DNase (TURBO DNA-free-kit; Thermo Fisher Scientific Inc., MA). cDNA was synthesized from the isolated RNA by RT-PCR (PrimeScript RT Reagent kit; TAKARA BIO Inc., Shiga, Japan). Quantitative real-time PCR was performed with SYBER Premix Ex Taq (TAKARA BIO Inc.) or SYBER Premix DimerEraser (TAKARA BIO Inc.) using a CFX384^TM^ Touch Real-time PCR Detection System (BioRad Laboratories, Inc., Hercules, CA). Relative gene expression was determined against HPRT gene expression. The following PCR primers were designed: forward primer 5′-ATACAGCTGCCGTGTTTCAG-3′ and reverse primer 5′-AGCCATCTTATCACCCAAGAA-3′ for *Il31* mRNA; and forward primer 5′-GGCCAGACTTTGTTGGATTTG-3′ and reverse primer 5′-CGCTCATCTTAGGCTTTGTATTTG-3′ for *Hprt* mRNA.

### Flow cytometry

Spleen cells were incubated with anti-mouse CD16/CD32 mAb (2.4G2; BD Biosciences, San Jose, CA) in FACS buffer (HBSS containing 2% FCS) for FcR blocking for 15 min on ice, and then incubated with BV421-conjugated anti-mouse CD3 mAb (145-2C11; Biolegend, San Diego, CA), APC-conjugated anti-mouse CD8 mAb (53-6.7; Biolegend), PE-conjugated anti-mouse CD4 (GK1.5 Biolegend) or Siglec F (E50-2440; BD Biosciences) mAb, PE-Cy7-conjugated anti-mouse CD19 mAb (6D5; Biolegend) or Gr1 (RB6-8C5; Affymetrix eBioscience, San Diego, CA) mAb for 20 min on ice. The expression profile of each cell surface marker on 7-aminoactinomycin D (7-AAD; Sigma-Aldrich Co. LLC, St. Louis, MO) -negative cells was analyzed on a MACSQuant Analyser (Miltenyi Biotec, Bergisch Gladbach, Germany) with MACSQuantify Software (Miltenyi Biotec) and FlowJo software (Tree Star Inc., Ashland, OR).

### Skin DC migration

Skin DC migration was determined as described elsewhere^28^. In brief, mice were epicutaneously treated with 40 µl of 0.5% (w/v) FITC isomer I (Sigma-Aldrich Co. LLC) in a 1:1 mixture of acetone (Wako Pure Chemical Industries, Ltd., Osaka, Japan) and dibutyl phthalate (Sigma-Aldrich Co. LLC) (20 µl to each surface of the left ear) and the vehicle alone (20 µl to each surface of the right ear). Twenty-four hours later, submaxillary lymph nodes (LNs) were separately collected from both the FITC-treated left and vehicle-treated right ears. After incubation with anti-mouse CD16/CD32 mAb (2.4G2, BD Biosciences) for 15 minutes on ice, LN cells were stained with PE-conjugated anti-mouse CD11c mAb (HL3, BD Biosciences) and APC-conjugated anti-mouse MHC Class II (I-A/I-E) mAb (M5/114.15.2, Affymetrix eBioscience) for 20 minutes on ice. The percentage of FITC-positive cells among 7-AAD-negative MHC Class II^hi^ CD11c^+^ cells was determined using a FACS Verse (BD Biosciences), and data analyses were performed using FlowJo software (Tree Star Inc.).

### Skin DC maturation

A shaved area on the back and both surfaces of both ears of mice were painted with 150 µl and 20 µl of 0.5% FITC isomer I, respectively. Twenty-four hours later, inguinal, axillary and submaxillary LNs were harvested and pooled. LN single-cell suspensions were prepared and incubated with anti-mouse CD16/CD32 mAb for 15 minutes on ice. The cells were then stained with APC-conjugated MHC-class II mAb, PE-Cy7-conjugated anti-mouse CD11c mAb, and PE-conjugated mAb for CD86 (GL-1, Biolegend), CD40 (3/23, Biolegend) or OX40L (RM134L, Biolegend) for 20 minutes on ice. The expression levels of CD86, CD40 and OX40L on 7-AAD-negative MHC Class II^hi^ CD11c^+^ FITC^+^ cells were determined using a FACS Verse (BD Biosciences), and data analyses were performed using FlowJo software (Tree Star Inc.).

### Induction of contact hypersensitivity (CHS)

CHS was induced with FITC and DNFB as described elsewhere^28,29^. Briefly, 2 days after shaving the back hair with electrical clippers, the back skin was treated with 150 µl of 1.0% FITC isomer I in a 1:1 mixture of acetone and dibutyl phthalate or 25 µl of 0.5% DNFB (SIGMA) in a 4:1 mixture of acetone and olive oil. Five days later, the respective mice were challenged with 40 µl of 0.5% FITC isomer I or 0.2% DNFB (each surface of the left ear) and 40 µl of the vehicle alone (each surface of the right ear). The ear thickness was measured before and after FITC challenge using dial thickness gauges (Ozaki MFG. Co., Ltd., Tokyo, Japan).

### Measurement of levels of serum immunoglobulins

Sera were collected from mice 4 days after FITC challenge during FITC-induced CHS. The levels of FITC-specific Igs in the sera were determined by ELISA as described elsewhere^29^. In brief, 96-well flat-bottom plates (Thermo Fisher Scientific Inc.) were coated with 20 µg/ml FITC-conjugated OVA at 4 °C overnight. After the wells were blocked with PBS containing 1% FBS, optimally diluted serum samples (IgG1 = 1/100; IgG2b = 1/100; IgG2c = 1/10) were placed in the wells, and the plates were incubated at room temperature for 2 hours. After washing, HRP-conjugated anti-mouse IgG1, IgG2b or IgG2c mAb (Bethyl Laboratories, Inc., TX) was added, followed by incubation at room temperature for 1 hour. For enzymatic reaction, TMB substrate (Nacalai Tesque Inc.) was used as a substrate. The reaction was stopped by addition of 0.2 M H_2_SO_4_, and then the absorbance (450 nm) was measured using a VersaMax (Molecular Devices, LLC, Sunnyvale, CA). Data show the absorbance value at 450 nm. The levels of total IgE in sera were determined using an ELISA kit (Bethyl Laboratories, Inc.) in accordance with the manufacturer’s instructions.

### FITC-specific LN cell responses

FITC-specific LN cell responses were determined as described elsewhere^28,29^. Briefly, mice were epicutaneously treated with 1.0% FITC isomer I in a 1:1 mixture of acetone and dibutyl phthalate on both the left and right ears (20 μl on one surface of each ear). Six days later, submaxillary LNs were collected. The LN cells were cultured in the presence and absence of 40 μg/ml FITC at 37 °C for 72 hours. The levels of cytokines in each culture supernatant were determined with mouse IFN-γ, IL-4 and IL-17A ELISA kits obtained from Biolegend (San Diego, CA) or Peprotech (Rocky Hill, NJ).

### Assessment of scratching behaviour

SCLABA-Real (NOVELTEC) was used to assess scratching behavior (frequency and duration) at 24 and 48 hours after challenge with FITC or DNFB during FITC- or DNFB-induced CHS.

### Histology

Twenty-four hours after FITC or vehicle challenge, the ear skins were harvested, fixed in Carnoy’s fluid and embedded in paraffin. Sections were prepared and stained with hematoxylin-eosin.

### Immunohistochemistry

Twenty-four hours after epicutaneous challenge with DNFB as described above, the ear skin was frozen in OCT compound (Sakura Finetek, Tokyo, Japan). Sections (4.5-μm thick) were prepared using a cryostat (Leica) and dried on aminosilane-coated slide glasses (Matsunami Glass, Osaka, Japan). The sections were fixed in 4% paraformaldehyde at room temperature for 10 min and incubated with Blocking One Histo (Nacalai Tesque, Inc.) and Avidin/Biotin Blocking Kit (Vector Laboratories, CA) at room temperature for 10 min. They were then incubated with primary Abs, rabbit anti-mouse CD3 Ab (ab5690; Abcam plc., Tokyo, Japan), hamster anti-mouse CD11c Ab (ab33484; Abcam plc.), rat anti-mouse F4/80 (MCA497GA; Bio-Rad), biotin-conjugated goat anti-mouse IL-31 Ab (BAF3028; R&D Systems), rat anti-mouse Ly-6G/-6C Ab (ab2557; Abcam plc.) or rabbit anti-mouse tryptase Ab (ab134932; Abcam plc.), at 4 °C overnight. After washing, the sections were incubated with secondary Abs/reagent, Alexa fluor^®^ 594-conjugated donkey anti-rabbit Ab (ab150064; Abcam plc.), Alexa fluor^®^ 594-conjugated donkey anti-rat Ab (ab150156; Abcam plc.), Alexa fluor^®^ 594-conjugated goat anti-hamster Ab (A21113: Thermo Fisher Scientific), or Alexa fluor^®^ 488-conjugated streptavidin (S11223: Thermo Fisher Scientific), at 4 °C for 60 min. Nuclei were stained with DAPI (R37606; Thermo Fisher Scientific). Images were acquired using a fluorescent microscope (BZX-700, KEYENCE, Tokyo, Japan) and analyzed with a software (BZ-analysis application, KEYENCE).

### Statistics

A two-tailed Mann-Whitney U test was performed for statistical significance using Graphpad Prism software (Graphpad Prism). P values of less than 0.05 were considered statistically significant.
